# Low SARS-CoV-2 seroprevalence in the Austrian capital after an early governmental lockdown

**DOI:** 10.1038/s41598-021-89711-5

**Published:** 2021-05-12

**Authors:** Marie-Kathrin Breyer, Robab Breyer-Kohansal, Sylvia Hartl, Michael Kundi, Lukas Weseslindtner, Karin Stiasny, Elisabeth Puchhammer-Stöckl, Andrea Schrott, Manuela Födinger, Michael Binder, Markus Fiedler, Emiel F. M. Wouters, Otto C. Burghuber

**Affiliations:** 1 Department of Respiratory and Critical Care Medicine, Clinic Penzing, Vienna, Austria; 2 Ludwig Boltzmann Institute for Lung Health, Vienna, Austria; 3 Sigmund Freud University, Faculty of Medicine, Vienna, Austria; 4 Center for Public Health, Medical University of Vienna, Vienna, Austria; 5 Center for Virology, Medical University of Vienna, Vienna, Austria; 6 Institute of Laboratory Diagnostics, Clinic Favoriten, Vienna, Austria; 7 Vienna Healthcare Group, Vienna, Austria; 8 Ludwig Boltzmann Institute for Lung Health, The Austrian LEAD Study, Klink Penzing, Gebäude G, 2. Stock, Sanatoriumstrasse 2, 1140 Vienna, Austria

**Keywords:** Immunology, Infectious diseases, Viral infection

## Abstract

We analyzed SARS-CoV-2 seroprevalence in a large, well-described representative Viennese cohort after an early governmental lockdown with respect to the occurrence of symptoms and household transmission. Participants of the LEAD Study, a population-based cohort study from Vienna, Austria, were invited along with their household members (April 20th to May20th 2020). Sera were analyzed using anti-SARS-CoV-2 immunoassay including a neutralization test as a confirmatory assay. A total of 12,419 individuals participated (5984 LEAD participants; 6435 household members), 163 (1.31%; 59 LEAD cohort members) of whom were SARS-CoV-2 antibody positive. The estimated number of COVID-19 cases projected from our findings by age and sex for Vienna was 21,504 (1.13%). Cumulative number of positively tested cases in Vienna until May 20th 2020 was 3020, hence 7.1 times (95% confidence interval 5.5–9.1) lower than projected. Relative risk (RR) of seropositivity by age was highest for children aged 6–9 years [RR compared to age group 20–49: 1.21 (CI 0.37–4.01)], lowest for ≥ 65 years [RR 0.47 (CI 0.21–1.03)]. Half of the positive individuals developed no or mild symptoms. In a multivariate analysis, taste and smell disturbances were most strongly related to SARS-CoV-2 positivity. Infection probability within households with one confirmed SARS-CoV-2-specific antibody-positive person was 31%. Although seroprevalence was very low (1.13%) for a central European capital city, due to an early governmental lockdown, SARS-CoV-2 infections were more prevalent than officially reported polymerase chain reaction-positive cases. Of note, seroprevalence was highest in young children. Half of SARS-CoV-2 antibody-positive subjects had no or only mild symptoms. Taste and smell disturbances were most prominent, possibly guiding clinicians in diagnosing SARS-CoV-2 infection.

## Introduction

Coronavirus disease 2019 (COVID-19), caused by severe acute respiratory syndrome coronavirus 2 (SARS-CoV-2), was first identified in January 2020 in Wuhan, China, and has since spread rapidly, with more than 90 million cases worldwide and about 1.9 million deaths by December 2020^1^. During the winter months, multiple countries worldwide were faced with a dramatic increase in SARS-CV-2 infection rates, requiring drastic public health measures to prevent healthcare systems’ exhaustion. During this process, it was of utmost importance to evaluate such measures’ effect and evaluate continuously speed of viral spread on a population level. In the face of the emerging of the massive third wave of the pandemic, with the need to implement partial or complete nationwide lockdowns, again, data on their effectiveness were crucial.

In addition to data obtained by molecular methods, seroepidemiological studies can quantify the proportion of infected individuals within a population independently of the occurrence of symptoms^2^. Multiple such seroprevalence studies have been performed (e.g., in the USA, Spain, and Switzerland), with seroprevalence rates of 3–10% during the early phase of the pandemic (until the third quarter of 2020)^3–6^. In Austria, however, SARS-CoV-2 initially spread from the western parts of the country to the eastern, with an early, complete nationwide governmental lockdown from March to May 2020, resulting in only 86,000 confirmed cases and approximately 1000 COVID-19 related deaths^7^.

In this study, we determined the seroprevalence in Vienna, Austria’s capital, located in the Eastern part of the country and with over 1.9 million inhabitants, to evaluate this first and early lockdown. To do this, we recruited more than 12,000 individuals, all participants of the LEAD (Lung, hEart, sociAl, boDy) cohort Study, a population-based epidemiological cohort representative of the Viennese general population aged 6–85 years, and assessed seroprevalence with an antibody assay and a neutralization test (NT) as a confirmatory assay. This seroprevalence analysis also provided data on risk factors for SARS-CoV-2 infection and household transmission (including children), on the clinical manifestation of confirmed SARS-CoV-2, and the correlation of NT titers with clinical severity.

## Methodology

### Study design

Sera for this LEAD COVID-19 Study (ClinicalTrials.gov; NCT04346264) were prospectively collected in Vienna, Austria from April 20th to May 20th 2020. The LEAD study cohort is a general population cohort providing a representative sample of the general Viennese population in terms of age, sex, smoking habits, and place of residence. Individuals from this cohort, as well as their household members, were invited by letter and offered COVID-19 antibody testing^8^.

At the single visit, participants completed a questionnaire capturing COVID-19 specific symptoms^9^. If symptoms were reported, contact with the COVID-19 hotline or general practitioner (GP), sickness leave, or hospital visits was recorded. Previous swab and/or antibody testing and their results were captured, along with any travel to areas with COVID-19 warnings. For all LEAD cohort members, demographic, socio-economic, and clinical parameters are available from the main study database; household members with serologically confirmed SARS-CoV-2 infection were asked for information on weight, height, comorbidities, and smoking status. All participants with SARS-CoV-2-specific antibodies were asked whether they had adhered to governmental quarantine rules (isolation, with no shared meals or bathrooms).

The study was approved by the local ethics committee (Ethics Commission of the City of Vienna, Austria, EK 20-082-VK), and participants signed informed consent; those for children aged under 18 years were signed by parents/legal representatives. All methods were performed in accordance with the relevant guidelines and regulations.

### SARS-CoV-2-specific antibody assays

#### First-line testing

For all participants, blood was collected from the vein (whole blood in a 5 mL separating gel tube) at the LEAD study center, which was centrifuged (2000*g* for 10 min), serum collected (removal of the supernatant in 250 µL aliquots into screwable cryotubes), and then frozen and stored at − 20 °C. Serum samples were analyzed at the Clinic Favoriten, Vienna, using a commercial electrochemiluminescence immunoassay (Elecsys^®^ Anti-SARS-CoV-2), on a Cobas e411 analyzer (both Roche, Mannheim, Germany) according to the manufacturer’s instructions. Sera with a cut off index ≥ 1.0 were considered reactive (indicating a previous infection).

#### Confirmatory assays

Reactive sera in the first-line testing were retested in an in-house NT and a commercial immunoassay, the Euroimmun SARS-CoV-2-IgG enzyme-linked immunosorbent assay (ELISA; Euroimmun, Lübeck, Germany) using the S1 domain of the viral spike protein as antigen, utilizing the manufacturer’s protocol and cut-off values. The NT used Vero E6 (ATCC^®^ CRL-1586) cells and live SARS-CoV-2 (GISAID/EPI_ISL_438123/hCoV-19/Austria/CeMM0360/2020). Two-fold serial dilutions of heat-inactivated serum samples were incubated with 50–100 TCID50 virus for 1 h at 37 °C, then added to cell monolayers and incubated 2–3 days at 37 °C. Virus neutralization was assessed by cytopathic effects (CPE), with NT titers expressed as the reciprocal of the serum dilution protecting against virus-induced CPE; values ≥ 10 were considered positive. Only when both assays tested positive was the positive antibody test result by first-line testing considered confirmed.

### Statistical analysis

Projection of SARS-CoV-2 positive cases in Vienna was based on age- and sex-specific confirmed presence of SARS-CoV-2-specific antibodies in the LEAD sample, weighted by population size, with 95% confidence intervals (CIs) from Poisson distribution. In a sensitivity analysis, invited members of the LEAD cohort with undelivered invitation letters were assigned the same fraction as those participating, assuming that persons not aware of the opportunity to get tested had the same rate of positivity as those included in the analyses; those that received the letters but did not attend were assigned 80% of the age- and sex-adjusted confirmed positivity rate, assuming that those that chose not to participate were less likely to believe they had been exposed to SARS-CoV-2, which predicts to some degree the likelihood of infection.

Possible risk factors for positivity were first analyzed by univariate logistic regression. Those factors statistically significant (5% level) were then analyzed by simultaneous multiple logistic regression, correcting for age, systemic low-grade inflammation and diabetes type 2. A similar procedure was used to assess the relationship between each reported symptom and antibody positivity. Symptoms were submitted to cluster analysis using the Lance & Williams similarity index and complete linkage as amalgamation rule.

COVID-19 severity was categorized as: very high, hospitalization; high, contact with a GP or sickness leave, and symptoms from at least three clusters; moderate, symptoms from two clusters; mild, symptoms from one cluster only; asymptomatic, no symptoms. For the computation of NT titer geometric means, values ≥ 80 were arbitrarily set to 160; those < 10 were set to 5.

The household transmission was analyzed with a mixed logistic model using the household as a random factor. Likelihood of transmission was determined as functions of attributes of the index case (the household case with earliest symptoms) and of household contacts. Analyses were performed using Stata 13.1 (StataCorp, College Station, TX, USA).

## Results

### Seroprevalence and projected number of infections

**Figure 1:**
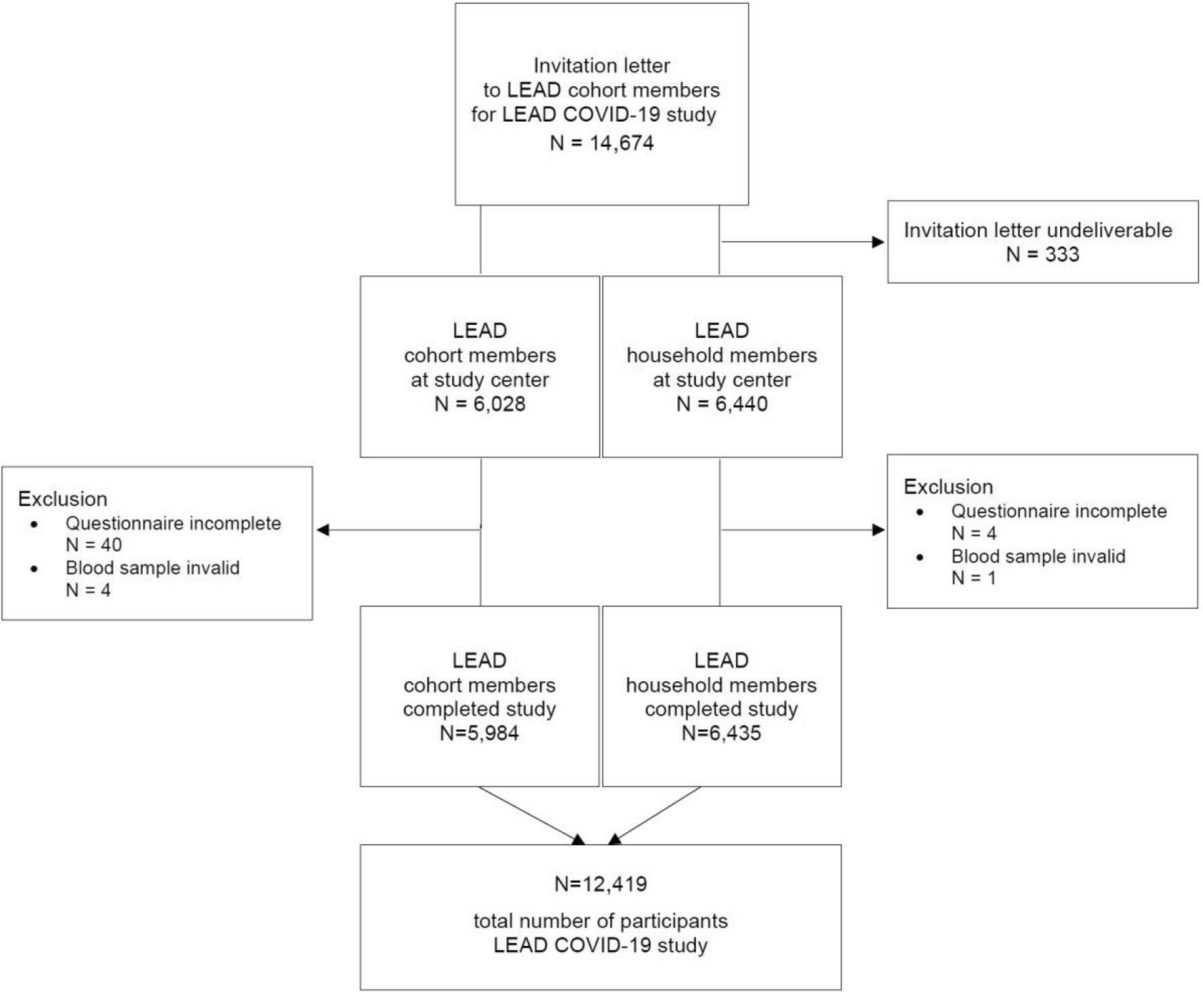
Flow chart of participants.

In total 12,419 subjects, 5984 from LEAD and 6435 household members, completed this study (Fig. 1). Household members were younger (43.4 ± 18.7 vs. 46.4 ± 20.4; p < 0.01) and less often female (54.3% vs. 56.1%; p = 0.0413) than the LEAD cohort members (Table 1). In the whole LEAD cohort and their household members, the positivity rate of SARS-CoV-2 specific antibodies was 1.31% (n = 163; 59 LEAD cohort and 104 household members). More household members had SARS-CoV-2-specific antibodies than LEAD cohort participants (1.6% vs. 1.0%; p < 0.0001).

**Table 1:** Comparison of LEAD cohort members and household members.

	LEAD cohort members	LEAD household members	p-value
	N = 5984	N = 6435	
Age, mean ± SD	46.4 ± 20.4	43.4 ± 18.7	< 0.0001
**Age groups (6–85 years), n (%)**			
6–9 years	184 (3.1%)	218 (3.4%)	< 0.0001
10–19 years	636 (10.6%)	662 (10.3%)	
20–49 years	2149 (35.9%)	2869 (44.6%)	
50–64 years	1756 (29.3%)	1854 (28.8%)	
65+ years	1259 (21.0%)	832 (12.9%)	
Sex, female, n (%)	3357 (56.1%)	3492 (54.3%)	0.0413
SARS-CoV-2 antibody positive, n (%)	59 (1.1%)	104 (1.6%)	0.0021
Travelling in area with warning, n (%)	219 (3.7%)	248 (3.9%)	0.6037
**Previous swab test, n (%)**	154 (2.6%)	258 (4.0%)	< 0.0001
Test positive	17 (11%)	27 (10.5%)	0.8700
**Previous antibody test, n (%)**	21 (0.4%)	22 (0.3%)	1.0000
Test positive	2 (9.5%)	5 (22.7%)	0.4121

Based on this, the projected number of SARS-CoV-2 infections for Vienna was 21,504 (1.13% [95% CI 16,524–27,513; 0.87–1.45%]) for the period until May 20th 2020. The cumulative number of reported cases in Vienna until May 20th was 3020. Hence, the projected number is 7.1 times (95% CI 5.5–9.1) higher than the reported numbers suggesting there was a large number of unidentified cases.

### Manifestation of symptoms

**Figure 2:**
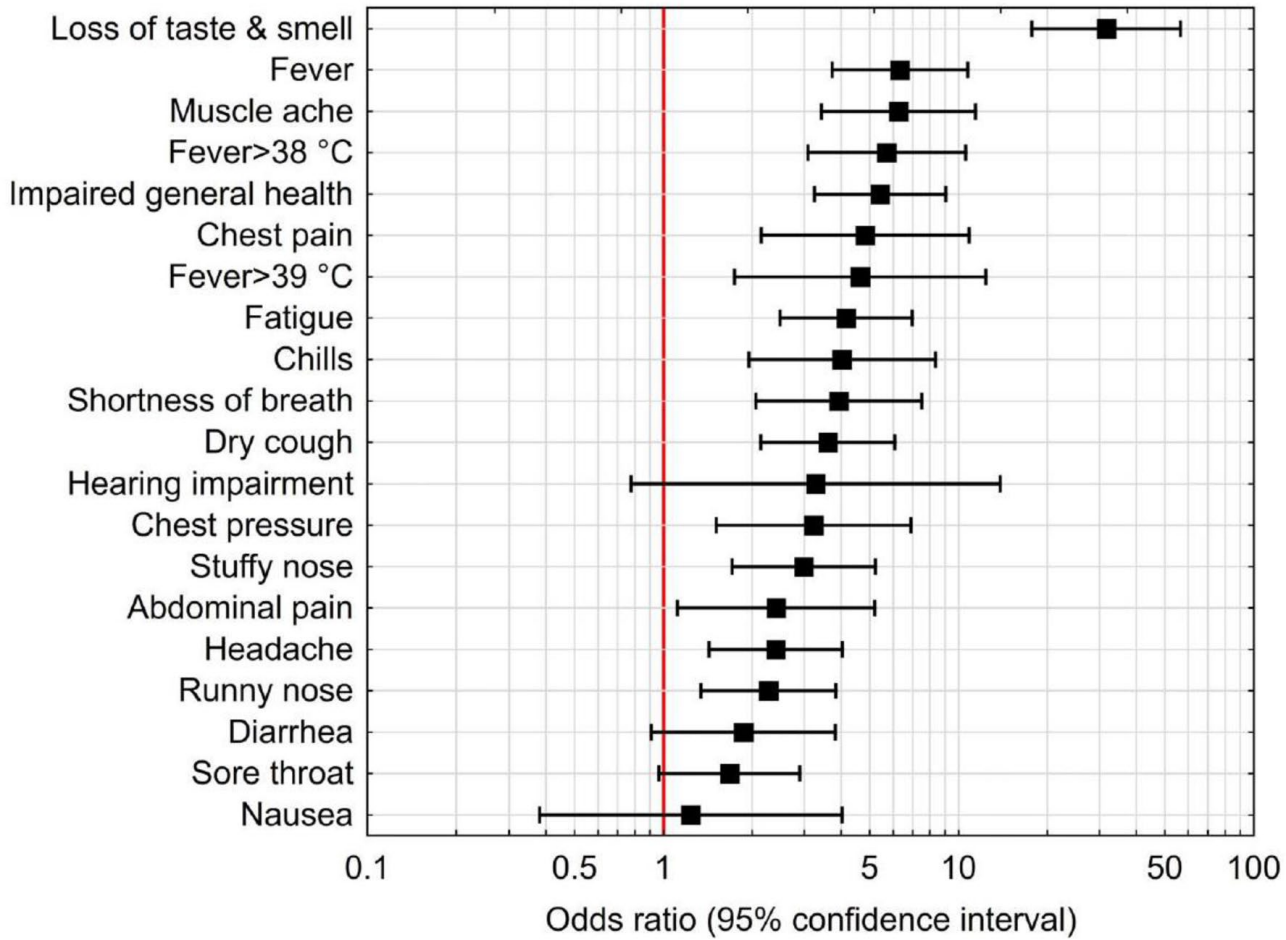
Relationship between symptoms and presence of SARS-CoV-2 specific antibodies in a multivariate analysis.

Univariate analyses of symptoms most frequently associated with the presence of SARS-CoV-2-specific antibodies are shown in Supplemental Table S1. In the multivariate analysis, the most frequent symptoms were impaired taste and smell (OR 31.66 [95% CI 17.75–56.46]; p < 0.0001), fever (6.33 [3.73–10.74]; p < 0.0001), muscle ache (myalgia; 6.26 [CI 3.44–11.41]; p < 0.0001), and impaired general health (5.42 [3.25–9.06]; p < 0.0001; Fig. 2 and Supplemental Table S1).

Significant predictors of SARS-CoV-2-specific seropositivity were loss of taste and smell, diabetes type 2, fever, and influenza vaccination within the previous 12 months. Systemic low-grade inflammation (high-sensitivity C-reactive protein [hs-CRP] > 3 mg/L) was a protective factor (Table 2). Of note, the highest relative risk of seropositivity was in children aged 6–9 years, with the lowest risk in those aged ≥ 65 years (Table 3), such that age by decade was a protective factor (Table 4). Other associated risk factors were diabetes type 2, previous swab test (regardless of outcome), and previous stay in an area with a SARS-CoV-2 related travel warning during the respective period (Table 4 and Supplemental Table S2).

**Table 2:** Stepwise analyses of symptoms and occurrence of SARS-CoV-2-specific antibody positivity.

	OR	95% CI	p-value
Loss of taste and smell	20.79	11.16–38.73	< 0.0001
Fever	3.49	1.95–6.26	< 0.0001
Diabetes type 2	9.22	2.57–33.01	0.0006
Influenza vaccination	3.04	1.61–5.74	0.0006
Chronic low grade inflammation (hs-CRP > 3 mg/L)	0.26	0.09–0.74	0.0116

*OR* odds ratio, *CI* confidence interval, *hs-CRP* high-sensitivity C-reactive protein.

**Table 3:** The relative risk of SARS-CoV-2-specific seropositivity by age.

	Antibody negative	Antibody positive	OR (95% CI)	p-value
6–9 years	181 (98.4%)	3 (1.6%)	1.21 (0.37–4.01)	0.7542
10–19 years	627 (99.2%)	5 (0.8%)	0.58 (0.22–1.51)	0.2674
20–49 years	2119 (98.6%)	27 (1.3%)	1 (ref)	
50–64 years	1736 (99.0%)	16 (1.0%)	0.76 (0.42–1.37)	0.3576
≥ 65 years	1250 (99.4%)	8 (0.6%)	0.47 (0.21–1.03)	0.0577

*OR* odds ratio, *CI* confidence interval.

**Table 4:** Association of risk factors with SARS-CoV-2-specific antibody positivity.

	Antibody negative	Antibody positive	OR	95% CI	p-value
Diabetes type 2	1.0%	4.8%	6.93	1.85–25.88	0.0040
Previous swab test performed	2.3%	27.0%	11.61	6.12–22.02	< 0.0001
Travelling in an area with warning	3.5%	15.9%	3.62	1.70–7.71	0.0008
Age by decade	46.5 (20.4)	40.7 (20.4)	0.84	0.71–0.99	0.0346
Systemic low-grade inflammation	21.4%	6.5%	0.29	0.10–0.83	0.0211

*OR* odds ratio, *CI* confidence interval.

**Figure 3:**
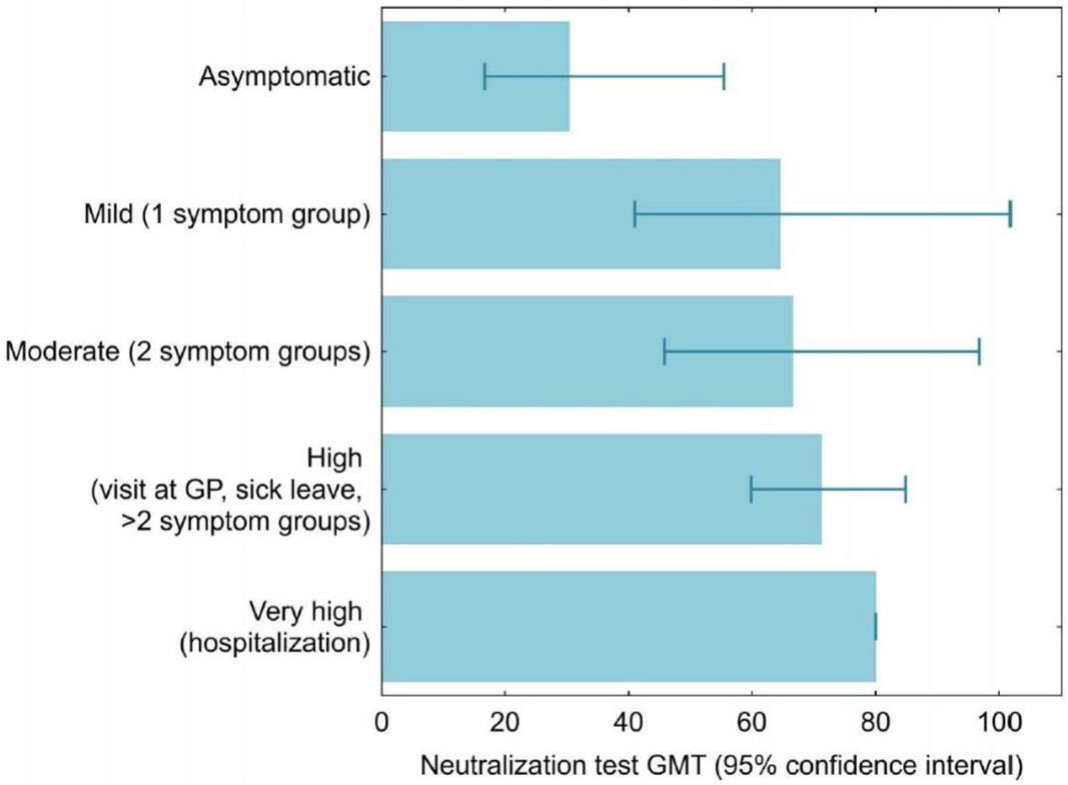
The severity of symptoms related to neutralization titer. *GMT* geometric mean titer, *GP* general practitioner.

Of those with SARS-CoV-2-specific antibodies and symptoms, 84.7% reported a loss of taste and smell, dry cough, fever, fatigue, or impaired general health (OR 13.04 [95% CI 8.50–20.01]; p < 0.0000). Interestingly, the severity of COVID-19-related symptoms correlated with the NT titers, being lowest for asymptomatic individuals (n = 6; titer median 30.4 [95% CI 16.7–55.4]), followed by mild (n = 9; 64.6 [CI 41.0–101.7]), moderate (n = 13; 66.6 [45.8–96.8]), and high symptom severity (n = 30; 71.3 [59.8–84.8]), with highest NT-titers in one hospitalized individual (titer > 80) (Fig. 3). When projected to the Vienna population, the six asymptomatic cases in our analyses equates to 1945 individuals, with the nine mild and 14 moderate cases equating to 2418 and 5355 individuals, respectively. The total asymptomatic rate was 20.8% (n = 17; LEAD, n = 6; household members, n = 11).

### Infections in household members

In 82 non-single households, we identified the index case (SARS-CoV-2-specific antibody-positive individual) having the earliest symptoms (Table 5). The total number of SARS-CoV-2 exposed household members in homes with at least one positive case was 280 (range 2–8 per household; median 3.4), 241 (86%) of whom took part in the study. Of the 159 cases at risk (241 exposed household members minus 82 index cases), 50 developed SARS-CoV-2-specific antibodies (31.4%), which is approximately 30 times higher than the general ambulatory infection risk (1%). Predictors of household transmission were age and NT titer, with the isolation of the index case reducing transmission risk. The mean number of persons in the household, presence of children, and sex of the index case did not significantly affect household transmission (Table 6 and Supplemental Tables S3 and S4). Of note, none of the asymptomatic index cases infected a household member.

**Table 5:** Household transmission rate by household size.

Number in household	Number of households	Participants n (%)	Antibody positive n (%)	Transmission n (rate)	Rate, all household members^a^
2	28	56 (100)	36 (64.3)	8 (0.29)	0.286
3	17	45 (88.2)	30 (66.7)	13 (0.46)	0.382
4	22	74 (84.1)	39 (52.7)	17 (0.33)	0.258
5	10	40 (80.0)	17 (42.5)	7 (0.23)	0.175
6	2	9 (75.0)	2 (22.2)	0	0.000
7	1	4 (57.1)	2 (50.0)	1 (0.33)	0.167
8	2	13 (81.3)	6 (46.2)	4 (0.36)	0.286
Total	82	241 (86.1%)	132 (54.8%)	50 (0.31)	0.253

^a^Assuming the members who did not participate were not positive.

**Table 6:** Predictors of risk of household transmission by attributes of the index case and household.

	Household transmission				
	No	Yes	OR	95% CI	p-value
Age^a^ Mean (SD)	39.2 (18.8)	49.4 (17.6)	1.36	1.13–1.65	0.0013
NT titer^b^ GMT (SF)	50.7 (2.0)	105.3 (1.8)	4.81	1.85–12.46	0.0012
Persons in the HH Mean (SD)	3.2 (1.3)	3.7 (1.6)	0.94	0.76–1.17	0.5850
Children in the household	21 (33.9)	18 (54.5)	1.65	0.85–3.22	0.1419
Sex, female	31 (49.2)	17 (51.5)	0.80	0.42–1.54	0.5086
Isolation	13 (20.6)	2 (6.1)	0.26	0.07–0.89	0.0322

*CI* confidence interval, *GMT* geometric mean titer, *NT* neutralization test, *SF* Scatter factor, *SD* standard deviation.

^a^Odds ratio (OR) by 10 years increase.

^b^OR by tenfold increase.

## Discussion

In this seroepidemiological study with over 12,000 participants of the Viennese population, we analyzed the SARS-CoV-2-specific seroprevalence after an early governmental lockdown and correlated the antibody positivity rate with the manifestation of symptoms and household transmission. As main results we demonstrate that the early lockdown in Austria resulted in a very low seroprevalence of 1.13% in Vienna (a European capital city with 1.9 million inhabitants) and that SARS-CoV-2 infections were 5.5–9.1 times more prevalent than officially reported numbers suggested. Furthermore, nearly half of SARS-CoV-2 antibody-positive cohort individuals showed no or only mild symptoms, with disturbance of smell and taste as the most prominent symptom. These data highlight the effectiveness of a complete lockdown as a public health measure to reduce the speed of viral spread, the extent of the SARS-CoV-2 infection rate that was not identified by molecular testing, and the potential utility of loss of smell and taste to clinically identify COVID-19.

An interesting aspect of our findings is that the projected number of cases for Vienna was 5.5–9.1 times larger than the reported numbers, although seroprevalence (1.13%) was considerably lower than in other seroprevalence studies performed in Europe during the same period (5–10% in Switzerland^5^ and 3.7–6.2% in Spain^6^). These data strongly indicate the effectiveness of a complete lockdown as a public health measure to reduce the speed of SARS-CoV-2 spread. In Austria, an early strict nationwide lockdown was initiated on March 16th 2020 when a total of only 151 cases were reported until that time point. This lockdown not only included mask requirements, school closures, and physical distancing, but also home isolation of multiple sectors of the population. The initial SARS-CoV-2 cases were reported in Tyrol in the western part of the country (related to the early spread of the virus in Italy), although the virus subsequently spread from the western to the eastern parts of the country. The nationwide lockdown on March 16th 2020 may thus have significantly reduced SARS-CoV-2 transmission in Vienna (in the eastern part of Austria), although it is the largest city, with 1.9 million inhabitants.

Of note, we found the lowest seroprevalence in persons aged ≥ 65 years, consistent with a previous study^6^, supporting the hypothesis that although older people are likely to have a more severe course when infected, such individuals are more careful in their daily lives and in maintaining social distancing. Importantly, our finding of the highest seroprevalence being in children aged 6–9 years is in line with the cross-sectional results from a Swiss study showing a seroprevalence of SARS-CoV-2 antibody-positive schoolchildren aged 6–9 years of about 3.8%, with lower seroprevalence in children aged 10–14 years of 2.5% and in those aged 14–16 years of 1.5%, indicating an inverse trend with age^10^. It is worth noting that the present study is population-based, and demonstrates that children play an import role in SARS-CoV-2 antibody seroprevalence, although younger age was not associated with increased household transmission.

In terms of symptoms, our data clearly illustrate that COVID-19 severity varies from very mild to severe. In particular, about half of the infected individuals reported multiple symptoms, contacted their general practitioner or were on sick leave, and one individual was hospitalized due to COVID-19. Most striking is the high prevalence of smell and taste disturbance in the seropositive participants. Thus, our study demonstrated that a combination of loss of taste and smell with dry cough and fever was highly discriminative for SARS-CoV-2 antibody positivity. A systematic review and meta-analysis, including 1627 participants, suggested a 36.6% prevalence of olfactory dysfunction using non-validated instruments, increasing to 86.6% using validated instruments^11^. The prevalence of gustatory dysfunction among COVID-19 patients in the review was 43.9%, with about 38% self-reporting loss of taste and/or smell. Furthermore, results from a UK observational cohort study revealed 78% of approximately 500 SARS-CoV-2-positive subjects had a loss of smell and/or taste^12^. For many patients with COVID-19, olfactory dysfunction may be the initial presenting symptom^13^. In our study, 38.1% of seropositive participants reported an impaired sense of taste or smell, with these symptoms only present in 1.9% of those seronegative (Supplemental Table S1). It is assumed that SARS-CoV-2 targets cells in the sinonasal tract and olfactory epithelium by binding to the angiotensin-converting enzyme (ACE)-2 receptor^14^, the highest levels of which are expressed in the goblet and ciliated cells in the nasal epithelium and sustentacular and basal cells. SARS-CoV-2 may invade the central nervous system through the olfactory bulb following intranasal infection^15^.

Asymptomatic transmission of SARS-CoV-2 is a substantial challenge for COVID-19 pandemic control. Indeed, in this study, 10.3% of the SARS-CoV-2-specific seropositive LEAD cohort and 10.5% of the infected household members reported no symptoms, with 39% reporting mild symptoms without seeking medical attention. Stringhini et al. reported a higher number of asymptomatic seropositive participants in a Swiss study: one third were asymptomatic with regional distribution of 21.9–35.8%^5^. Asymptomatic infections have been reported as an important factor in outbreaks of COVID-19 in nursing facilities^16^, with a median duration of viral shedding of 19 days and low IgG and neutralizing antibody levels in the early convalescent phase^17^. These unique characteristics of SARS-CoV-2 argue for broad testing including asymptomatic individuals, with the use of face masks as measures to combat this pandemic.

A specific fraction of individuals infected with SARS-CoV-2 are more likely to become ill than others, with mortality significantly higher in males and those with comorbid cardiovascular disease, hypertension, diabetes, respiratory disease or cancers^18^. In our population of detailed phenotyped cohort members, diabetes type 2 and previous influenza vaccination were important risk factors for being infected. We hypothesize that previous influenza vaccination reflects an overall complex risk profile in our cohort, since vaccinated individuals were older, and more often had respiratory and cardiovascular comorbidities. Remarkably, the presence of low-grade systemic inflammation, manifested by elevated CRP levels was identified as a protective factor. CRP, a short pentraxin, belongs to the group of acute-phase proteins, a family of highly conserved proteins involved in the host defense against infections. The capacity of acute-phase proteins to limit viral replication and spread within the host as part of an early defense response before the induction of specific antibody response is widely recognised^19^. This possible protective role to COVID-19 protection needs further exploration. Our data, however, do not confirm an increased risk of smoking or hypertension on SARS-CoV-2 transmission. Previous epidemiological studies have shown no significant association between current smoking and severe COVID-19^20,21^. A systematic review, however, concluded that smoking may be associated with disease progression and adverse outcomes^22^. Recent data suggest upregulation of the ACE-2 receptor may be mediated by nicotine-related effects on the nicotine acetylcholine receptor alpha-7 subtype in airway epithelial cells; further studies using alpha 7-nAChR antagonists are needed to unravel the link between COVID-19 and smoking^23^.

In the present study, the probability of a serologically confirmed SARS-CoV-2 individual infecting a household contact was 31%, approximately 30 times higher than the general ambulatory infection risk. For comparison, household transmission rates have been reported of 67.6% in a Swiss study^5^, 16.3% in one study in China^24^, and 30% in a second Chinese study^25^, with the differences possibly explained by the level of individual quarantine. In the present study, we identified various predictors of household transmission, with a higher relative risk with increasing age of the index case confirming the general assumption that older subjects are more vulnerable to a SARS-CoV-2 infection. Of note, children in the same household had no particular predictive or preventive role concerning the transmission of SARS-CoV-2, which can be explained by similar virus concentrations as compared to adults with COVID-19^26^.

Finally, our study identified an association between clinical COVID-19 severity and the absolute levels of neutralizing antibodies, confirming previous findings^27^. Furthermore, our study suggests that the level of neutralizing antibody titers was also associated with the rate of transmission to household contacts. In support of this, none of the asymptomatic index cases, having the lowest NT titers, infected a household member. In contrast, fever, dry cough, and loss of taste and smell were associated with increased risk of SARS-CoV-2 infection, and patients with these symptoms also displayed higher NT titers. A possible explanation could be that higher virus concentrations in symptomatic patients cause a higher grade of contagiousness, a stronger humoral immune response and higher antibody levels, although this requires confirmation in future studies.

Our study's key strength is the representative participation of over 12,000 individuals in a seroprevalence study from a well-described epidemiological cohort aged 6–85 years, and their household members, allowing an in-depth analysis of familial transmission. Of note, we also used an NT as a confirmatory serological assay. A weakness of our study is that participants were invited via letter rather than randomly sampled, potentially leading to an overestimation of individuals who already suspected a previous infection and were more willing to participate.

In conclusion, our study provides evidence that an early and complete lockdown in Austria led to a very low seroprevalence rate in the capital with 1.9 million inhabitants, indicating the high efficacy of this rigorous public health measure. Our study furthermore, confirms previous reports that SARS-CoV-2 infection is more prevalent than the observed numbers suggest, although suggesting a significantly higher underreporting than previously estimated. We found that disturbance of smell and taste is the most prominent symptom of SARS-CoV-2 infection, possibly guiding clinicians in diagnosing the infection. Finally, our data indicate a high probability of transmission within household contacts, and no lower SARS-CoV-2 seroprevalence in children from 6 to 9 years of age as compared to adults.

## Supplementary Information

Supplementary Tables.

## Data Availability

Data can be obtained on specific request.
