# Supplementary material for: Low SARS-CoV-2 seroprevalence in the Austrian capital after an early governmental lockdown
Source: Sci Rep. 2021 May 12;11:10158. doi: 10.1038/s41598-021-89711-5 (PMC8115109; doi:10.1038/s41598-021-89711-5)
Supplement: Supplementary file 1 — Supplementary Tables. [file 41598_2021_89711_MOESM1_ESM.docx]

**Supplement**

**Low SARS-CoV-2 seroprevalence in the Austrian capital after an early governmental lockdown**

Marie-Kathrin Breyer^1,2*^ | Robab Breyer-Kohansal^1,2^ | Sylvia Hartl^1,2,3^ | Michael Kundi^4^ | Lukas Weseslindtner^5^ | Karin Stiasny^5^ | Elisabeth Puchhammer^5^ | Andrea Schrott^2^ | Manuela Födinger^3,6^ | Michael Binder^7^ | Markus Fiedler^2^ | Emiel FM Wouters^2^ | Otto C Burghuber^2,3^.

**S Table 1. Relationship between symptoms and presence of SARS-CoV-2-specific antibodies in univariate and multivariate analysis sorted by increasing univariate p-value.**

|  |  |  | Univariate | | | Multivariate^1^ | | |
| --- | --- | --- | --- | --- | --- | --- | --- | --- |
|  | Antibody  negative | Antibody  positive | OR | 95% CI | p Fisher | OR | 95% CI | p Wald |
| Impaired sense of taste or smell | 1.9% | 38.1% | 31.18 | 18.15-53.58 | <0.0001 | 31.66 | 17.75-56.46 | <0.0001 |
| Fever | 9.1% | 42.9% | 7.51 | 4.52-12.46 | <0.0001 | 6.33 | 3.73-10.74 | <0.0001 |
| Impaired general health | 13.1% | 47.6% | 6.05 | 3.67-9.97 | <0.0001 | 5.42 | 3.25-9.06 | <0.0001 |
| Fatigue | 13.2% | 46.0% | 5.61 | 3.40-9.25 | <0.0001 | 4.16 | 2.48-6.99 | <0.0001 |
| Dry cough | 14.1% | 42.9% | 4.58 | 2.77-7.58 | <0.0001 | 3.61 | 2.14-6.08 | <0.0001 |
| Muscle ache (myalgia) | 5.1% | 25.4% | 6.39 | 3.58-11.40 | <0.0001 | 6.26 | 3.44-11.41 | <0.0001 |
| Fever >38C | 4.9% | 23.8% | 6.12 | 3.39-11.07 | <0.0001 | 5.71 | 3.09-10.55 | <0.0001 |
| Stuffy nose | 11.7% | 33.3% | 3.77 | 2.22-6.40 | <0.0001 | 2.99 | 1.71-5.23 | 0.0001 |
| Shortness of breath (dyspnea) | 5.8% | 20.6% | 4.22 | 2.27-7.84 | 0.0001 | 3.93 | 2.06-7.50 | <0.0001 |
| Headache | 20.3% | 41.3% | 2.76 | 1.67-4.58 | 0.0002 | 2.40 | 1.43-4.04 | 0.0010 |
| Chills | 4.0% | 15.9% | 4.48 | 2.25-8.92 | 0.0002 | 4.03 | 1.94-8.34 | 0.0002 |
| Runny nose | 18.4% | 36.5% | 2.54 | 1.52-4.27 | 0.0009 | 2.27 | 1.34-3.85 | 0.0024 |
| Chest pain | 2.6% | 11.1% | 4.63 | 2.07-10.31 | 0.0015 | 4.82 | 2.14-10.85 | 0.0001 |
| Feeling pressure on the chest | 4.4% | 14.3% | 3.65 | 1.78-7.48 | 0.0018 | 3.23 | 1.51-6.90 | 0.0025 |
| Fever >39C | 1.4% | 7.9% | 6.18 | 2.42-15.82 | 0.0020 | 4.64 | 1.74-12.38 | 0.0022 |
| Sore throat | 19.1% | 31.7% | 1.96 | 1.15-3.35 | 0.0157 | 1.67 | 0.97-2.90 | 0.0662 |
| Abdominal pain | 5.2% | 12.7% | 2.67 | 1.26-5.66 | 0.0163 | 2.41 | 1.12-5.19 | 0.0252 |
| Diarrhea | 7.9% | 15.9% | 2.19 | 1.11-4.33 | 0.0322 | 1.87 | 0.91-3.83 | 0.0887 |
| Impaired sense of hearing | 1.1% | 3.2% | 3.08 | 0.74-12.89 | 0.1467 | 3.28 | 0.78-13.82 | 0.1057 |
| Nausea/vomiting | 3.6% | 4.8% | 1.33 | 0.41-4.26 | 0.5000 | 1.24 | 0.38-4.03 | 0.7229 |

OR: odds ratio; CI: confidence interval; ^1^Age, systemic low-grade inflammation, diabetes type 2, influenza vaccination within 12 month considered.

**S Table 2. Association of risk factors with SARS-CoV-2-specific antibody positivity.**

|  | Antibody  negative | Antibody  positive | OR | 95% CI | p-Fisher |
| --- | --- | --- | --- | --- | --- |
| Age | 46.5 (20.4) | 40.7 (20.4) | 0.84 | 0.71-0.99 | 0.03464 |
| Sex (female) | 56.3% | 44.4% | 0.66 | 0.39-1.11 | 0.11407 |
| Socio economic status (highest) | 52.9% | 51.6% | 1.02 | 0.66-1.59 | 0.92070 |
| Coronary artery disease (myocardial infarction or angina pectoris) | 8.3% | 11.1% | 1.63 | 0.68-3.91 | 0.27627 |
| Peripheral artery occlusion disease, stroke, or carotid calcification | 7.1% | 9.5% | 1.80 | 0.71-4.59 | 0.21619 |
| Hypertension | 22.0% | 14.3% | 0.58 | 0.24-1.40 | 0.22628 |
| Cancer | 7.0% | 4.8% | 0.84 | 0.25-2.85 | 0.78393 |
| Allergy | 42.0% | 41.3% | 0.91 | 0.55-1.53 | 0.73468 |
| Adiposity (BMI≥30 kg/m²) | 17.8% | 12.7% | 0.91 | 0.39-2.11 | 0.81878 |
| Smoking, yes | 20.1% | 25.4% | 1.45 | 0.80-2.64 | 0.22526 |
| Liver dysfunction (GOT>40 U/L) | 5.0% | 3.2% | 0.67 | 0.16-2.80 | 0.58188 |
| Systemic low-grade inflammation (hs CRP>3 mg/L) | 21.4% | 6.5% | 0.29 | 0.10-0.83 | 0.02110 |
| Hyperlipidemia (Chol>250 mg/dL or LDL>200 mg/dL) | 17.0% | 8.1% | 0.60 | 0.23-1.55 | 0.29098 |
| Respiratory dysfunction (FEV_1_ or TLC < LLN) | 10.6% | 9.5% | 0.97 | 0.41-2.30 | 0.95324 |
| Diabetes type 2 | 1.0% | 4.8% | 6.93 | 1.85-25.88 | 0.00401 |
| Influenza vaccination within 12 month | 15.8% | 27.0% | 2.56 | 1.40-4.68 | 0.00218 |

OR: odds ratio; CI: confidence interval; BMI: body mass index; GOT: glutamic oxaloacetic transaminase; hs CRP: high sensitive C-reactive protein; Chol: Cholesterol; LDL: Low-density lipoprotein; FEV_1_: forced expiratory volume in the first second; TLC: total lung capacity; LLN: lower limit of normal.

**S Table 3. Relationship between household transmission (SARS-CoV-2 antibody positivity of a household member after the index case) and risk factors of the index case.**

|  | Antibody  negative | Antibody  positive | OR | 95% CI | p Fisher |
| --- | --- | --- | --- | --- | --- |
| Age ≥65 years | 6.6% | 14.0% | 2.30 | 0.81–6.54 | 0.1398 |
| Sex (female) | 51.5% | 46.0% | 0.80 | 0.42–1.54 | 0.6200 |
| Coronary artery disease (myocardial infarction or angina pectoris) | 3.0% | 2.0% | 0.67 | 0.07–6.13 | 1.0000 |
| Peripheral artery occlusion disease, stroke, or carotid calcification | 4.4% | 8.0% | 1.87 | 0.50–6.92 | 0.4629 |
| Hypertension | 12.6% | 10.0% | 0.77 | 0.27–2.21 | 0.7997 |
| Diabetes type 2 | 5.9% | 4.0% | 0.67 | 0.14–3.25 | 1.0000 |
| Cancer | 3.7% | 6.0% | 1.67 | 0.38–7.27 | 0.4451 |
| HIV | 0.7% | 1.0% | 1.35 | 0.04–40.87 | 1.0000 |
| Allergy | 32.4% | 44.0% | 1.64 | 0.85–3.19 | 0.1675 |
| Liver dysfunction (GOT>40 U/L) | 9.1% | 5.0% | 0.50 | 0.02–10.25 | 1.0000 |
| Obesity (BMI >30kg/m²) | 7.5% | 12.0% | 1.68 | 0.58–4.89 | 0.3811 |
| Renal dysfunction (eGFR ≤60ml/min/1.73m²) | 0.0% | 0.0% |  |  |  |
| Inflammation (hs CRP>3 mg/L) | 0.0% | 0.0% |  |  |  |
| Hyperlipidemia (Chol>250 mg/dL or LDL>200 mg/dL) | 4.5% | 22.2% | 6.00 | 0.72–49.84 | 0.1294 |
| Respiratory dysfunction (FEV_1_ or TLC < LLN) | 13.6% | 20.0% | 1.58 | 0.27–9.32 | 0.6317 |
| Smoking, yes | 20.0% | 14.0% | 0.65 | 0.26–1.61 | 0.3999 |
| Influenza vaccination within last 12 month | 18.4% | 18.0% | 0.97 | 0.42–2.26 | 1.0000 |

OR: odds ratio; CI: confidence interval; HIV, human immunodeficiency virus; BMI: body mass index. eGFR, estimated glomerular filtration rate; hs CRP: high sensitive C-reactive protein; GOT: glutamic oxaloacetic transaminase; Chol: Cholesterol; LDL: Low-density lipoprotein; FEV_1_: forced expiratory function in the first second; TLC: total lung capacity; LLN: lower limit of normal.

**S Table 4. Relationship between household transmission (SARS-CoV-2 antibody positivity of a household member after the index case) and risk factors and symptoms of the index case.**

|  | Antibody  negative | Antibody  positive | OR | 95% CI | p Fisher |
| --- | --- | --- | --- | --- | --- |
| Fever | 36.0% | 66.0% | 3.45 | 1.74-6.82 | 0.0004 |
| Fever>39C | 7.4% | 12.0% | 1.72 | 0.59-5.00 | 0.3766 |
| Fever>38C | 30.1% | 42.0% | 1.68 | 0.86-3.28 | 0.1604 |
| Chills | 21.3% | 42.0% | 2.67 | 1.33-5.36 | 0.0084 |
| Dry cough | 39.7% | 58.0% | 2.10 | 1.09-4.05 | 0.0310 |
| Shortness of breath | 16.2% | 34.0% | 2.67 | 1.27-5.61 | 0.0138 |
| Wheezing | 3.7% | 8.0% | 2.28 | 0.59-8.85 | 0.2532 |
| Sore throat | 28.7% | 28.0% | 0.97 | 0.47-1.99 | 1.0000 |
| Runny nose | 30.9% | 10.0% | 0.25 | 0.09-0.67 | 0.0039 |
| Stuffy nose | 27.9% | 18.0% | 0.57 | 0.25-1.28 | 0.1874 |
| Impaired general health | 41.9% | 68.0% | 2.95 | 1.48-5.84 | 0.0017 |
| Fatigue | 36.8% | 60.0% | 2.58 | 1.33-5.01 | 0.0072 |
| Muscle ache | 25.7% | 40.0% | 1.92 | 0.97-3.81 | 0.0706 |
| Loss of taste and smell | 39.0% | 56.0% | 1.99 | 1.03-3.84 | 0.2333 |
| Chest pain | 4.4% | 20.0% | 5.42 | 1.85-15.83 | 0.0019 |
| Chest pressure | 14.7% | 12.0% | 0.79 | 0.30-2.10 | 0.8123 |
| Headache | 36.0% | 44.0% | 1.40 | 0.72-2.70 | 0.3949 |
| Nausea | 5.1% | 16.0% | 3.51 | 1.20-10.26 | 0.0290 |
| Diarrhea | 11.0% | 14.0% | 1.31 | 0.50-3.44 | 0.6114 |
| Abdominal pain | 12.5% | 18.0% | 1.54 | 0.64-3.71 | 0.3466 |
| Visit at general practitioner | 19.1% | 50.0% | 4.23 | 2.10-8.52 | 0.0001 |
| Sickness leave | 26.5% | 44.0% | 2.18 | 1.11-4.29 | 0.0315 |
| COVID-19 hotline contacted | 19.9% | 50.0% | 4.04 | 2.01-8.1 | 0.0001 |

OR: odds ratio; CI: confidence interval.
